# Risk Factors, Clinical Features, and Outcomes of Acute Pancreatitis in Children in Endemic Zone of Ascariasis in Eastern Bihar: A Hospital-Based Study

**DOI:** 10.7759/cureus.26177

**Published:** 2022-06-21

**Authors:** Piyali Bhattacharya, Manish Kumar, Anamika Kumari, Sudesh Kumar

**Affiliations:** 1 Department of Pediatrics, Mata Gujri Memorial Medical College, Kishanganj, IND

**Keywords:** endemic, acute, children, ascariasis, pancreatitis

## Abstract

Objective

This study aims to describe the etiology, clinical features, and outcomes of acute pancreatitis (AP) in children in an endemic area of hepatobiliary ascariasis.

Methods

This retrospective observational study included acute pancreatitis (AP) patients in the age group of 2-15 years from January 2019 to January 2022. Demographic profile, risk factors, clinical features, laboratory imaging, and outcome data were collected and analyzed.

Results

A total of 40 patients comprising of 21 males (52.5%) and 19 females (47.5%) were included. The median age of the diagnosis of AP was 8.3 years (range: 4-14 years). Biliary ascariasis was the most frequent etiology of AP (n=18, 45%), followed by gallbladder (GB) stone (n=6, 12%), trauma (n=1, 2.5%), hepatitis (n=1, 2.5%), valproate drug (n=1, 2.5%), and GB sludge (n=1, 2.5%). In clinical features, all cases had pain in the abdomen (n=40, 100%), followed by fever (n=9, 22.5%), nausea/vomiting (n=33, 82.5%), jaundice (n=2, 5%), and anemia (n=2, 5%). Three (7.5%) patients developed complications such as shock, pseudocyst, and necrotizing pancreatitis, respectively. The average median hospital stay was nine days (range: 4-20 days). No mortality occurred in our study.

Conclusions

This study revealed a high prevalence (12-13 cases/year) of AP in children in this area. Biliary ascariasis (45%) emerged as the commonest risk factor. Most of the cases suffered from mild AP (92%) and so recovered completely without any complication.

## Introduction

Acute pancreatitis (AP) is defined as reversible inflammation of the parenchyma of the pancreas, characterized by interstitial edema, acute inflammatory infiltrates, and different degrees of cellular apoptosis, necrosis, and hemorrhage [1]. AP manifests as acute onset of abdominal pain, vomiting, and nausea and is increasingly being recognized in children secondary to increased awareness. The International Study Group of Pediatric Pancreatitis in Search for a Cure (INSPPIRE) defines AP as the presence of any two of the following: acute abdominal pain consistent with AP, serum amylase and/or lipase levels more than or equal to three times the upper limit of normal, and imaging findings characteristic of AP [2].

The common cause of pancreatitis in children differs from that in adults. The common etiology of AP in the pediatric age group is biliary tract disease, adverse drug reactions, systemic disease, and trauma (reviewed in Bai et al.) [3]. Recently, several single-centered studies in the United States and other countries have reported an increase in the number of cases of AP in the pediatric age group [4-9]. Because of the increased prevalence of AP in our pediatric department, we attempted to study the various risk factors, clinical profiles, and outcomes of AP cases.

This area is a highly endemic zone for parasitic infestation, which is associated with hepatobiliary ascariasis and abdominal pain, leading to pancreatitis. Therefore, we compared the frequency of various etiologies, demographic factors, and outcomes of AP in children.

The objective of this study was to describe the etiology, clinical features, and outcomes of acute pancreatitis in children in areas endemic to hepatobiliary ascariasis.

## Materials and methods

This was a hospital record-based cross-sectional study conducted in Mata Gujri Memorial (MGM) Medical College and Lions Seva Kendra (LSK) Hospital, Kishanganj, Bihar. Prior approval was obtained from the institutional ethics committee, and consent was obtained from the medical superintendent.

Study duration

Data mining was conducted from January 2019 to January 2022 and analyzed from March 1, 2021, to January 2022.

Study population

A total of 180 children between two and 15 years of age who presented in the abovementioned period with acute abdominal pain in the pediatric emergency department with no previous documentation of specific causes of pain, chronic, or previous episodes of pancreatitis were included in the study. The diagnosis of acute pancreatitis was established using the INSPPIRE criteria [1].

Clinical definition of acute pancreatitis in children (INSPPIRE criteria)

Acute pancreatitis requires two or more of the three following criteria: abdominal pain suggestive of or compatible with AP, serum amylase and/or lipase activity more than or equal to three times the upper limit of normal, and imaging findings characteristic of or compatible with AP.

Data collection procedure

Data were collected by the primary investigator and coinvestigators. Data collection was done manually by reviewing the medical record sheet and with the help of the “Ayushman Bharat Unit” of the institute that provided ICD-11 coding for all the cases as DC 31.2.

Forty cases of AP diagnosed as per the INSPPIRE criteria were studied in detail with respect to the demographic profile, risk factors, clinical manifestations, outcomes, and complications.

Baseline laboratory (complete blood count, renal function tests, liver function tests, serum electrolyte, serum amylase, serum lipase, and random sugar) and radiological (abdominal USG and CECT) findings were also retrieved. Causes and complications were assessed based on clinical, laboratory, and radiological findings.

The following were considered as the primary outcome variables: etiologies, clinical features, and outcomes of acute pancreatitis in children in the endemic zone of hepatobiliary ascariasis.

In this retrospective data mining analysis, data were collected by a trained data collector who was trained by and under the supervision of the primary investigator who was not blinded to the study objective. The following data points were collected: age, sex, clinical features (fever, abdominal pain, nausea/vomiting, jaundice, and anemia), history of gallstones, passage of worms in stool, abdominal trauma, drug history, history of hepatitis, any fever with rash, parotitis, and family history of pancreatitis. The following test results were collected: amylase, lipase, SGPT, SGOT, RBS, and abdominal USG and CECT. The duration of the hospital stay was also assessed.

We planned to develop etiological categories starting with gallstone, biliary ascariasis, trauma, drug, viral infection, congenital anomaly of the pancreas, comorbidity, and idiopathic. Other categories were determined in a post hoc manner. Descriptive statistics were used for statistical analysis.

Data analysis

Baseline demographics are presented for a period of three years. Risk factors, clinical features, and outcomes were tabulated. Data analysis was performed using the Epi info software.

## Results

Among 180 children aged between two and 15 years presenting with acute abdominal pain in the pediatrics emergency department, with no specific cause or previous episodes of pancreatitis, 40 (22%) were diagnosed as cases of AP according to the INSPPIRE criteria and were included in this study. Among the cases, 21 (52.5%) were male and 19 (47.5%) were female. The median age at the diagnosis of acute pancreatitis was 8.3 years (range: 4-14 years) (Table 1).

**Table 1:** Summary of etiologies

Etiology (n (%))	Male (n (%))	Female (n (%))	0-5 years (n (%))	6-15 years (n (%))	Total (n (%))
Idiopathic	5 (41.6)	7 (58.33)	4 (33.3)	8 (66.6)	12 (30)
Gallstone	2 (33.33)	4 (66.66)	0 (0)	6 (100)	6 (15)
Biliary ascariasis	13 (72)	5 (27.77)	5 (27.77)	13 (72)	18 (45)
Trauma	1 (100)	0 (0)	0 (0)	1 (100)	1 (2.5)
Hepatitis	0 (0)	1 (100)	1 (100)	0 (0)	1 (2.5)
Drug-induced	1 (2.5)	0 (0)	0 (0)	0 (0)	1 (2.5)
Gallbladder sludge	0 (0)	1 (2.5)	0 (0)	0 (0)	1 (2.5)

Risk factors

Biliary ascariasis was the most frequent risk factor (n=18, 45%), followed by gallbladder (GB) stones (n=6, 12%), trauma (n=1, 2.5%), hepatitis (n=1, 2.5%), drugs (n=1, 2.5%), and GB sludge (n=1, 2.5%). In 12 (30%) cases, no definite risk factor was associated (Table 2).

**Table 2:** Main radiographic findings of acute pancreatitis in pediatric cases (N=40) USG: ultrasonography; CECT: contrast-enhanced computed tomography; CBD: common bile duct; GB, gallbladder; MPD, main pancreatic duct

Radiographic findings (n (%))	USG (N=40)	CECT (N=37)
Normal	11 (27.5)	0 (0)
Enlarged, bulky pancreas	17 (42.5)	13 (32.5)
CBD worm	12 (30)	8 (20)
GB stone	5 (12.5)	4 (10)
MPD worm	4 (10)	2 (5)
Peripancreatic edema	1 (2.5)	0 (0)
Necrotizing pancreatitis	0 (0)	1 (2.5)
Choledocholithiasis	1 (2.5)	2 (5)
Pseudocyst	1 (2.5)	1 (2.5)

Clinical presentation and investigations

Abdominal pain was present in all the cases, with other presentations being fever (n=9, 22.5%), nausea/vomiting (n=33, 82.5%), jaundice (n=2, 5%), and anemia (n=2, 5%). Comorbidity or clinical history of patients with AP included passage of worms in stool (n=18, 45%), gallstones (n=6, 15%), abdominal trauma (n=1, 2.5%), hepatitis (n=1, 2.5%), and infection (n=1, 2.5%).

All patients underwent abdominal USG, in which 11 (27.5%) patients showed normal findings and the rest showed enlarged, bulky pancreas (n=17, 42.5%), CBD worm (n=12, 30%), GB stone (n=5, 12.5%), MPD worm (n=4, 10%), peripancreatic edema (n=1, 2.5%), necrotizing pancreatitis (n=1, 2.5%), choledocholithiasis (n=2, 5%), and pseudocyst (n=1, 2.5%).

Abdominal CECT was done in 37 patients, which provided similar significant findings (Table 3). In three patients, CECT was not performed as they refused to provide consent for the same.

**Table 3:** Baseline demographics

	Male (N=21, 52.5%) (n (%)	Female (N=19, 47.5%) (n (%)	Total (N=40) (n (%)
Mean age (years)	8.05	8.7	8.375
Clinical presentation			
Abdominal pain	20 (50)	19 (47.5)	40 (100)
Fever	4 (44.44)	5 (55.55)	9 (22.5)
Nausea/vomiting	17 (51.51)	16 (48.48)	33 (82.5)
Jaundice	1 (50)	1 (50)	2 (5)
Anemia	1 (50)	1 (50)	2 (5)
Clinical history (%)			
Gallstone	2 (33.33)	4 (66.66)	6 (15)
Passage of worm in stool	13 (72)	5 (27.77)	18 (45)
Abdominal trauma	1 (100)	0 (0)	1 (2.5)
Hepatitis	0 (0)	1 (100)	1 (2.5)
Infection	0 (0)	1 (100)	1 (2.5)
Complications (%)			
Shock	1 (100)	0 (0)	1 (2.5)
Pseudocyst	1 (100)	0 (0)	1 (2.5)
No systemic complication	0 (0)	0 (0)	0 (0)
Average hospital stay (days)	9.4	10.1	9.75

Serum amylase and lipase levels were estimated in 39 patients, and one patient’s report was missing. According to the INSPPIRE criteria, the diagnosis of AP was >3 times the upper range of the standard value. Twenty-seven (67.23%) cases fulfilled the criteria for the diagnosis of AP, while 12 (30.7%) cases did not. Serum lipase levels were increased to >3 times the upper range in 26 (66.6%) cases, while in 13 (33.3%) cases, it was below the given criteria. The serum lipase level was >7 times the upper range in two (5.12%) cases.

Outcome

One patient developed shock as a complication, and pseudocyst and necrotizing pancreatitis were observed in two other cases. The median length of stay (LOS) was nine days (range: 4-20 days). In the preschool group and school-going and adolescent groups, the ranges were 4-13 and 6-20 days, respectively. None of the patients died of AP.

## Discussion

In the past decade, the epidemiology and risk factors of AP in children have been greatly developed [10-12]. The present study aimed to describe a baseline for AP in children in the northeastern part of India by defining the etiological factors, clinical presentation, hospital stay, and imaging findings among all cases of primary disease identified at our institute. AP in children was diagnosed at a rate of 12-13 cases per year in the present study, which is very high compared to a recent study conducted by Alabdulkareem et al. where the annual rate of the occurrence of AP was 2-3 cases on an average [13].

Risk factors

The risk factors for AP in children are variable. In an All India Institute of Medical Sciences (AIIMS)-based study published in 2003, Das et al. studied 54 children with pancreatitis, in which 28 were suffering from AP, and found the following as the common causes of AP: idiopathic (35.7%), drugs (14.3%), trauma (14.3%), cholelithiasis (10.7%), and hepatitis (3.6%) [14]. Poddar et al. studied 320 children with AP in India in 2017 and found that trauma (21%) and biliary tract diseases (10%) were the most common causes of AP in children [15]. Zhong et al. (2021), from China, studied 130 children with AP and found that biliary tract diseases (31%) and idiopathic (28%) were the most common risk factors [16]. In another study of 115 children in the United States published in 2019, idiopathic (31%) and drugs (23%) were the most common risk factors.

In our study, we found that biliary ascariasis (45%), idiopathic (30%), GB stones (12%), and trauma (2.5%) were the most common risk factors for AP in children. Biliary ascariasis was very high because of the high endemicity of ascariasis in this area. The other findings were consistent with those of the above studies. One (2.5%) case of AP was associated with the intake of Valparin. The diagnosis of idiopathic AP was not reassuring because the lack of genetic screening and ERCP prevented ruling out genetic and/or congenital anomalies.

Clinical features and investigations

Most patients in our study complained of abdominal pain (100%) at presentation associated with nausea/vomiting (82.5%) and fever (22.5%). Children with AP also had a history of passage of worms in stools (45%), GB stones (15%), trauma, and infection (2.5%). Park et al. (2010) in their study showed that the clinical presentation of AP in older children was commonly abdominal pain (91%) and nausea/vomiting (74%) [17]. Javid et al. (2013) showed that abdominal pain (94%), vomiting (76%), and nausea (20%) were the common clinical presentations [9]. The clinical presentation in this study was consistent with that of the aforementioned study.

The diagnosis of AP in our study was made when serum amylase levels were elevated >3 times the upper range (27 (67.23%) cases) and serum lipase levels were elevated (26 (66.6%) cases). The serum lipase level was >7 times the upper range in two cases (5.12%). Therefore, other criteria are required for the diagnosis of AP in pediatric patients. Coffey et al. (2013) suggested that serum lipase obtained within 24 hours of presentation and marker >7 times the upper limit of normal predicted severe AP and severe course of the disease [18].

Abdominal USG was performed in all patients (N=40), and abdominal CECT was performed in 37 cases. USG was normal in 27.5% of the cases and provided significant findings in 72.5% of the cases, including an enlarged, bulky pancreas (42.5%), CBD worm (30%), MPD worm (10%), and GB stone (12.5%). Abdominal CECT revealed significant findings in all cases (100%), in which enlarged, bulky pancreas and CBD were the most common findings. One case of necrotizing pancreatitis was also diagnosed by abdominal CECT. Uc et al. (2019) found that USG detected abnormal findings in approximately 50% of the patients, with an enlarged pancreas being the most frequent finding [19].

Outcomes

AP can be classified as mild, moderate, or severe according to the North American Society for Pediatric Gastroenterology, Hepatology, and Nutrition Pancreas Committee recommendation [20]. Mild AP is defined as AP without organ failure or local or systemic complications and usually resolves in the first week. Moderate AP, which shows the presence of transient organ failure, local complications, or exacerbation of morbidity, recovers in less than 48 hours. Severe AP is defined as persistent organ failure in >48 hours. Several scoring systems have been described to predict severity in pediatric patients [18,21]. In our study, three cases developed complications: one shock (2.5%), one pseudocyst (2.5%), and one necrotizing pancreatitis (2.5%). Most of the cases were mild AP (92.5%), moderate AP was observed in 7.5% of the cases, and no severe AP was documented. The median hospital stay was nine days (range: 6-20 days). No mortality was noted. In other studies, the mortality rate of pediatric AP was <5%, which was significantly lower than that of adult AP [20]. Pohl et al. (2015) reported that children with AP are hospitalized for 5-8 days, while infants and toddlers spend approximately 20 days in the hospital [22]. Gay et al. showed that the median LOS was four days (interquartile range: 3-7 days) [23]. Pancreatic pseudocysts may occur in approximately 15% of children with AP, and most cases resolve without intervention [24]. A subset of children with AP (6%-25%) may develop severe pancreatic inflammation, necrosis, and multiorgan failure (shock, renal failure, and pulmonary failure) [25,26].

## Conclusions

In our institution, the annual occurrence of 12-13 cases of pediatric AP on an average is high compared to the rest of India. Biliary ascariasis (45%) and idiopathic (30%) were found as the most common risk factors for pancreatitis in this area. Most patients had mild AP (92.5%) and recovered completely. Of the cases suffering from AP, 45% had a history of the passage of worms in the stool. The high prevalence of AP in this remote pediatric facility warrants early diagnosis and treatment so that mortality and morbidity can be reduced. Worm infestation, which was found to be the most commonly identified risk factor, can be prevented by increasing awareness of sanitation and drug prophylaxis programs.

The limitation of this study was that it was a retrospective, single-center study. The sample size was small; therefore, the findings of this study cannot be generalized. In this study, the idiopathic causes of pancreatitis (30%) were very high, which may be due to the lack of genetic screening and ERCP. The study data provided a foundation for prospective multicentered studies to increase our understanding of the epidemiology of the disease and establish a significant relationship between worm infestation as a risk factor and the increased prevalence of pancreatitis.
